# Operando Studies of the Electrochemical Dissolution of Silver Nanoparticles in Nitrate Solutions Observed With Hyperspectral Dark-Field Microscopy

**DOI:** 10.3389/fchem.2019.00912

**Published:** 2020-01-17

**Authors:** Kevin Wonner, Christian Rurainsky, Kristina Tschulik

**Affiliations:** Chair of Analytical Chemistry II, Faculty of Chemistry and Biochemistry, Ruhr University Bochum, Bochum, Germany

**Keywords:** dark-field microscopy, silver nanoparticle, hyperspectral dark-field microscopy, operando spectroscopy, plasmon resonance, single entity electrochemistry

## Abstract

Since nanoparticles are frequently used in commercial applications, there is a huge demand to obtain deeper insights into processes at the nanoscale. Especially, catalysis, chemical and electrochemical reaction dynamics are still poorly understood. Thus, simultaneous and coupled opto-and spectro-electrochemical dark-field microscopy is used to study *in situ* and *operando* the electrochemically driven dissolution mechanism of single silver nanoparticles in the presence of nitrate ions as non-complexing counter-ions, herein. Hyperspectral imaging is used to probe the intrinsic localized surface plasmon resonance of individual silver nanospheres before, during and after their electrochemical oxidation on a transparent indium tin oxide (ITO) electrode. Furthermore, optical video imaging was performed for additional information. Based on the complete loss of spectral information and intensity, a dissolution of the particles during the reaction was concluded. This way it is revealed that the dissolution of individual particles proceeds over several seconds, indicating a hindrance by the nitrate ions. Only electrochemical analysis does not provide this insight as the measured current does not allow distinguishing between successive fast dissolution of one particle after another or slow dissolution of several particles in a concerted manner. For comparison, experiments were performed in the presence of chloride ions. It was observed that the silver chloride formation is an instantaneous process. Thus, it is possible to study and define the reaction dynamics on the single nanoparticle level in various electrochemical systems and electrolyte solutions. Accordingly, *operando* opto- and spectro-electrochemical studies allow us to conclude, that the oxidation of silver to solvated silver cations is a kinetically slow process, while the oxidation to silver chloride is fast. We propose this approach as a new method to study electrocatalyst materials, their transformation and degradation under *operando* conditions.

## Introduction

Nanomaterials, like small nanoparticles (NP), show a high surface area to volume ratio and an altered electronic structure, as compared to their bulk counterparts (Kreibig, 1974; Zhu et al., 2013). Due to the tunable and often advantageous characteristics of those nano-entities they are widely used for industrial application (Cheng and Compton, 2014; Sokolov et al., 2015). For instance, silver nanoparticles (AgNPs) are applied in products like mobile phones, refrigerators, energy storage and batteries or in cosmetics, medicine and sportswear due to their antibacterial and anti-inflammatory properties (Cheng et al., 2013; Chernousova and Epple, 2013; Tschulik et al., 2013; Yang et al., 2013).

Catalytic processes in heterogeneous catalysis occur at the surface of the catalyst material. Especially, noble metal nanoparticles are often used as electrocatalyst and due to their high surface energies only a small amount of material is needed (Welch and Compton, 2006; Salehi-Khojin et al., 2013; Cao et al., 2015; Wang et al., 2015; Strickler et al., 2017; Konopatsky et al., 2018; El Arrassi et al., 2019; Evers et al., 2019). Electrochemical reductions and oxidations can be triggered in a deliberate way by application of a defined potential at the electrode and thus, the surface of the catalyst material (Shan et al., 2012). Even though nanoparticles, and in particular AgNPs, were intensively investigated with electrochemical and chemical methods, there is still a lack of knowledge about their behavior in solution, reactivities, and reaction mechanisms (Wong et al., 2009; Ngamchuea et al., 2018). Often, catalytic reaction pathways are also only poorly understood. Therefore, there is a huge demand to get deeper insights into reaction mechanisms, especially for electrochemically (and thus potential) driven reactions, at and of nanostructures on the single entity nanomaterial level. The latter one is of special interest, because even small changes in the size and shape of the materials (which likely alters by the synthesis) strongly influence and vary their electrochemical and physical behavior (Roduner, 2006; Viñes et al., 2014; Khan et al., 2017). For those purposes additional *operando* analytical, optical and spectroscopic methods are needed to get supplementary and time-dependent information to the electrochemical current signals.

Today, a huge variety of analytical methods is established, which are capable of studying the structure of nanomaterials *ex situ* under harsh conditions, like vacuum. Examples for such devices are scanning electron microscopy (SEM), transmission electron microscopy, scanning tunneling microscopy and atomic force microscopy (Binnig and Rohrer, 1983; Wang, 2000; Ferreira et al., 2015). Those methods offer a precise analysis (up to atomic resolutions) of shapes and sizes of nanostructures. However, they do not allow studying their real behavior in solution. In contrast to that it is also possible to use *in situ* methods, like dynamic light scattering or UV/VIS spectroscopy (Haiss et al., 2007; Amendola and Meneghetti, 2009; Ramos, 2017). Although it is feasible to measure nano-entities within their dispersion media, obtainable results are inaccurate and provide only imprecise size distributions. Moreover, it is only possible to guess the shapes by the evaluation of measured data. Instead of this, electrochemical methods can be used to study even single particles quantitatively during an electrochemical reaction *in situ* in various aqueous electrolytes (nanoparticle impact) (Tschulik et al., 2014). If a NP is in contact with a polarized electrode, it can undergo several chemical reactions, like complete conversion of the particle itself (Batchelor-McAuley et al., 2015; Saw et al., 2018) or catalytically enhanced reactions at the particle surfaces (Cheng and Compton, 2014).

Although particles sizes (Batchelor-McAuley et al., 2015), compositions (Saw et al., 2016), reaction kinetics (Saw et al., 2017), reaction layer distances (Little et al., 2018), or diffusion coefficients (Saw et al., 2018) can be determined by NP impacts their reactions mechanism and additionally processes at the surfaces are today poorly understood and still under debate. Despite working with similar experimental conditions, different results were obtained. Thus, various mechanisms of electrochemical single nanoparticle conversion were suggested (Cheng and Compton, 2014; Oja et al., 2017; Robinson et al., 2017; Saw et al., 2017; Ustarroz et al., 2017; Kanokkanchana et al., 2018). Since electrochemistry is limited by the amount of charge or current as the information that can be obtained from the measurements, additional non-electrochemical methods are needed to gain supplementary information. For instance, spectroscopy can be implemented and coupled with electrochemical methods to get *in situ* and *operando* information of a nanoparticle during its reaction.

**Figure 1:**
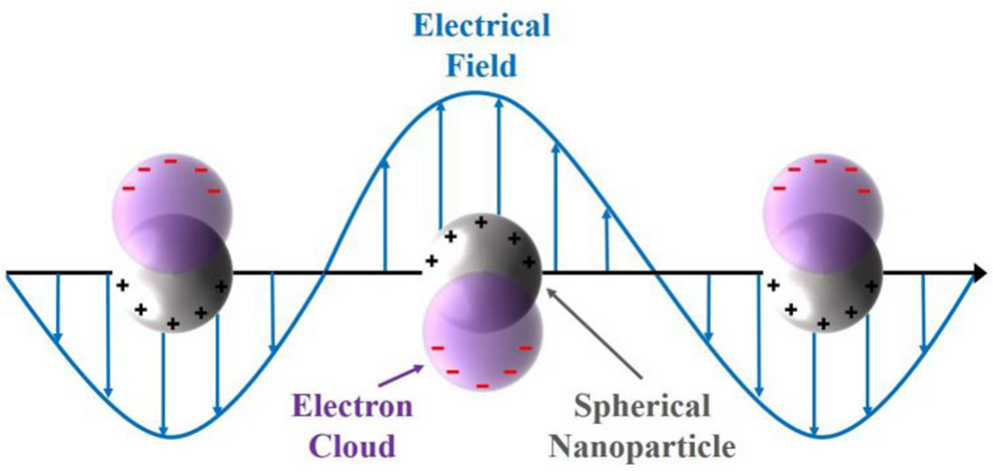
Representation of the localized surface plasmon resonance of a spherical nanoparticle and the electron cloud displacement due to the interaction with an incoming electric field.

A huge variety of those opto- and spectro coupled electrochemical methods has been reported in literature, like surface-enhanced Raman spectroscopy (Sherry et al., 2005; Stiles et al., 2008; Weber et al., 2015), electrogenerated chemiluminescence-imaging (Wilson et al., 2015) or single-particle fluorescence microscopy (Hao et al., 2017; Tanner et al., 2017). Metal nanoparticles, like silver and gold, exhibit an intrinsic localized surface plasmon resonance (LSPR), which is defined by the confinement of the size and the resulting electronic structure (Wilson and Willets, 2016). A representative illumination of the LSPR of a spherical nanoparticle is shown in Figure 1. The confined electrons in the conduction band, the electron cloud, are displaced by an incoming electric field (light) (Wilson and Willets, 2016). The negatively charged electron cloud is withdrawn again, due to the *Coulomb* forces of the remaining fixed and positively charged nuclei (Willets et al., 2017). Based on this coherent oscillation, incoming light with a larger wavelength compared to the size of the noble metal nanoparticles interact with those particles. Thus, light is scattered dependent on the incoming wavelength in relation to the size of the particle. Moreover, the LSPR depends on the shape, dielectric environment (surrounding media) and particle composition (Mock et al., 2002; Kelly et al., 2003; Wilson and Willets, 2016; Willets et al., 2017).

The excitation energy and intensity of the localized surface plasmon resonance of nanomaterials can be probed with plasmon-based electrochemical current microcopy (PECM) and particularly with dark-field microscopy (DFM). The latter is able to precisely study the spectral positions and intensities of the extinction spectra (LSPR) of individual nanoparticles by hyperspectral imaging (HSI) (Willets and van Duyne, 2007; Batchelor-McAuley et al., 2014; Fang et al., 2014; Brasiliense et al., 2016; Sundaresan et al., 2018; Sun et al., 2018; Wonner et al., 2018, 2019; Qiu et al., 2019). In previous works of the groups of *Tao* (Fang et al., 2014), *Kanoufi* (Batchelor-McAuley et al., 2014; Brasiliense et al., 2016), and our group (Wonner et al., 2019) silver nanoparticles were dissolved electrochemically in the presence of thiocyanate anions with PECM and DFM, respectively. A drawback of the PECM is that only intensities can be tracked during the reaction, whereas hyperspectral DFM can observe the spectral changes during a particle reaction. Moreover, previous works showed that it is even possible to resolve the spectral changes in the scattering spectrum of a single particle during an electrochemical oxidation by a composition change of the particle itself (Wonner et al., 2018).

Unexpectedly, although nearly the same electrochemical conditions are used for the oxidation of silver nanoparticles in the presence of nitrate, there is still controversy about the oxidation mechanism (Cheng and Compton, 2014; Patel et al., 2015; Oja et al., 2017; Saw et al., 2017; Ustarroz et al., 2017). On the one hand, the oxidation is described as an instantaneous and full dissolution, while on the other hand an incomplete oxidation with a continuous dissolution mechanism has been reported. For this purpose, hyperspectral dark-field microscopy (HSI-DFM) should be employed to study the dissolution process for detailed insight into the reaction dynamic of small nano-entities.

**Figure 2:**
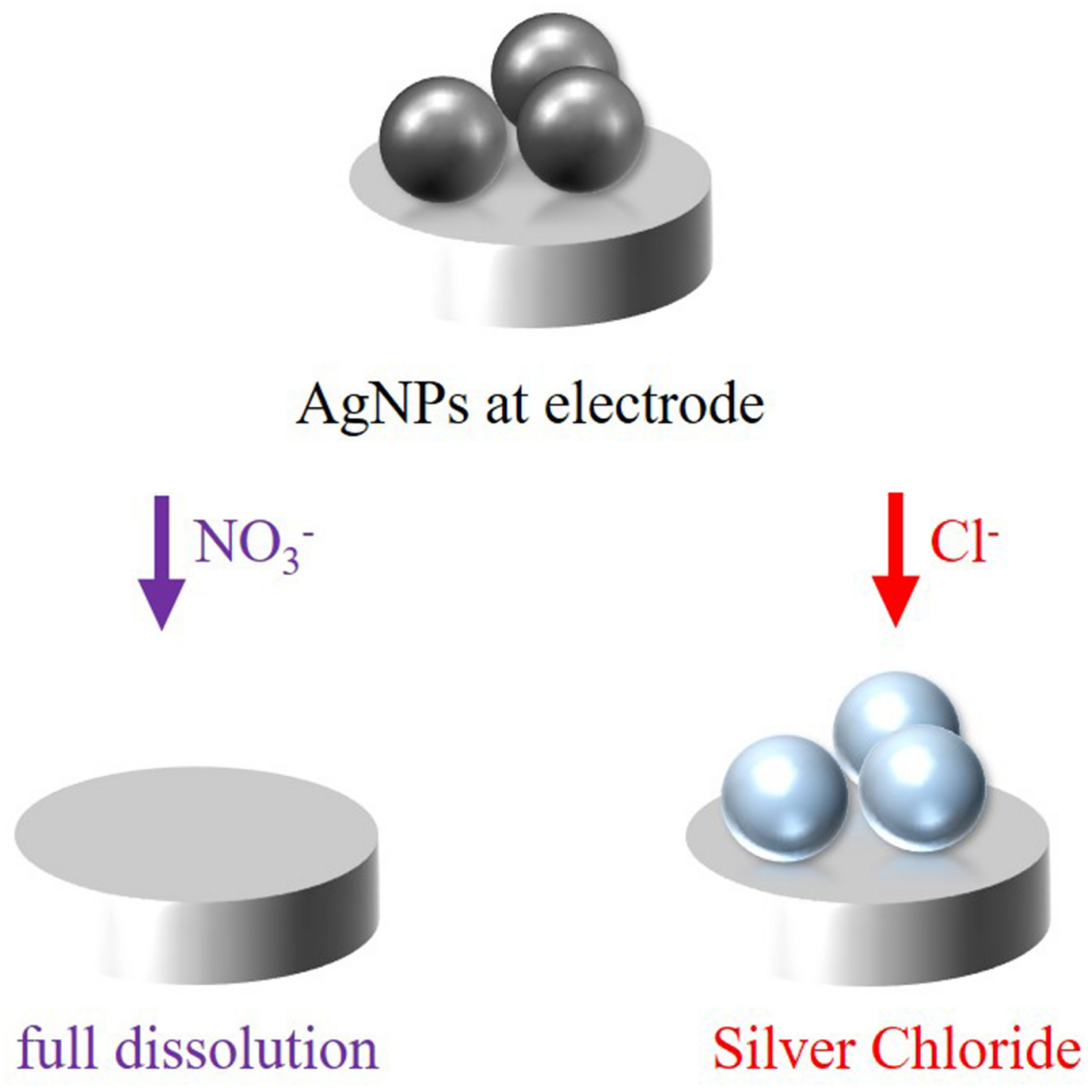
Schematic representation of the electrochemical oxidation of silver nanoparticles in the presence of either nitrate or chloride anions at a polarized electrode.

In this work, the oxidation process of silver nanoparticles is studied to figure out the mechanism of the oxidation in the presence of nitrate anions (${\text{NO}}_{3}^{-}$) of the electrolyte solution, which is illustrated in Figure 2. Moreover, the experiments should additionally be carried out in the presence of chloride (Cl^−^) anions to compare the reaction dynamics in the presence of two different anions (Figure 2), a complexing and a non-complexing anion. For reactions in chloride solution, an instant oxidation, and thus color change is expected with a defined spectral shift. For the two electrolytes, the reaction is given in Equation (1) and (2), respectively.

(1) $$\begin{array}{r} Ag\left(s\right)+N{O_{3}}^{-}\left(aq\right)⇌Ag^{+}\left(aq\right)+e^{-}+N{O_{3}}^{-}\left(aq\right) \end{array}$$

(2) $$\begin{array}{r} Ag\left(s\right)+Cl^{-}\left(aq\right)⇌AgCl\left(s\right)↓+e^{-} \end{array}$$

In the presence of non-complexing nitrate ions, silver will be oxidized to silver cations (Ag^+^), which are dissolved in the electrolyte solution (Equation 1). Hence, a decrease in intensity and loss of spectral information is expected during the oxidation of the particles. From the dissolution rate, conclusions about the mechanism of the electrochemical oxidation can be drawn. In contrast, the formation of silver chloride (AgCl) is thermodynamically driven in the presence of chloride ions, which favors the oxidation of the silver nanoparticles and shifts it to lower potentials (Equation 2) (Toh et al., 2013; Saw et al., 2018). The reaction is supposed to proceed in a fast way, which can be observed by an instantaneous spectral change. Moreover, an intensity alteration of the scattered light is supposed to happen, too (Brasiliense et al., 2018).

## Experimental Part

### Synthesis of Silver Nanoparticles

Fifty milliliters of ultra-pure water were heated in a 100 mL round bottom flask until boiling. Five hundred microliters of 10 mM AgNO_3_ (*Alfa Aesar*, 99.9995% metal basis) solution followed by 500 μL of a 60 mM tri-sodium citrate (*AnalR NORMAPUR, VWR chemicals*) solution were quickly added under vigorous stirring. The mixture was boiled under reflux for 1 h and no color change was observed. After that, first 500 μL of the 60 mM tri-sodium-citrate solution and subsequently 2 mg D-(+)-glucose (*AnalR NORMAPUR, VWR chemicals*) were added to the mixture. The mixture was boiled and stirred for another 1 h. Afterwards it was cooled down to room temperature using an ice bath and later centrifuged for 20 min at 20,000 rcf and the supernatant solution was removed until about 200 μL were left, the suspension was then refilled using ultra-pure water to 2 mL. The obtained particle suspension exhibited a concentration of roughly 0.25 μg/μL. The particle size was determined with underpotential deposition of lead on silver. A size distribution of 28 ± 3 nm was obtained (Grasmik et al., 2018). The used citrate served as a reducing and capping agent to prevent particle agglomeration. The electrochemical response of silvernanoparticles is not measurably affected by this small capping agent. Hence, this effect can be neglected.

### Chemicals and Materials

UV-treated ultrapure water (*Thermo Scientific Barnstead GenPure xCAD Plus*), exhibiting a conductivity of 0.055 μS cm^−1^ at 23°C, was used for all experiments, aqueous solutions and cleaning processes. All used chemicals were of analytical grade. Potassium chloride (KCl) and potassium nitrate (KNO_3_) with a purity of 99.0–100.5 and 99.999%, respectively, were purchased from *Sigma-Aldrich*. Used indium tin oxide (ITO) coated glass slides were purchased from *Sigma-Aldrich* and exhibited a surface resistivity of 8–12 Ω/sq. The size of the ITO-squares was 4 mm^2^. Gold was sputtered from a Ø57 × 0.1 mm gold target disc with a purity of 99.999%, which was purchased from *Micro to Nano*. All glassware and ITO-glass slides were sonicated for 30 min in a mixture of 50% acetone, 25% ethanol and 25% isopropanol. Afterwards, the procedure was repeated in pure water. All glassware was dried with an argon stream.

### Electrochemical Cell and Dark-Field Microscopy

**Figure 3:**
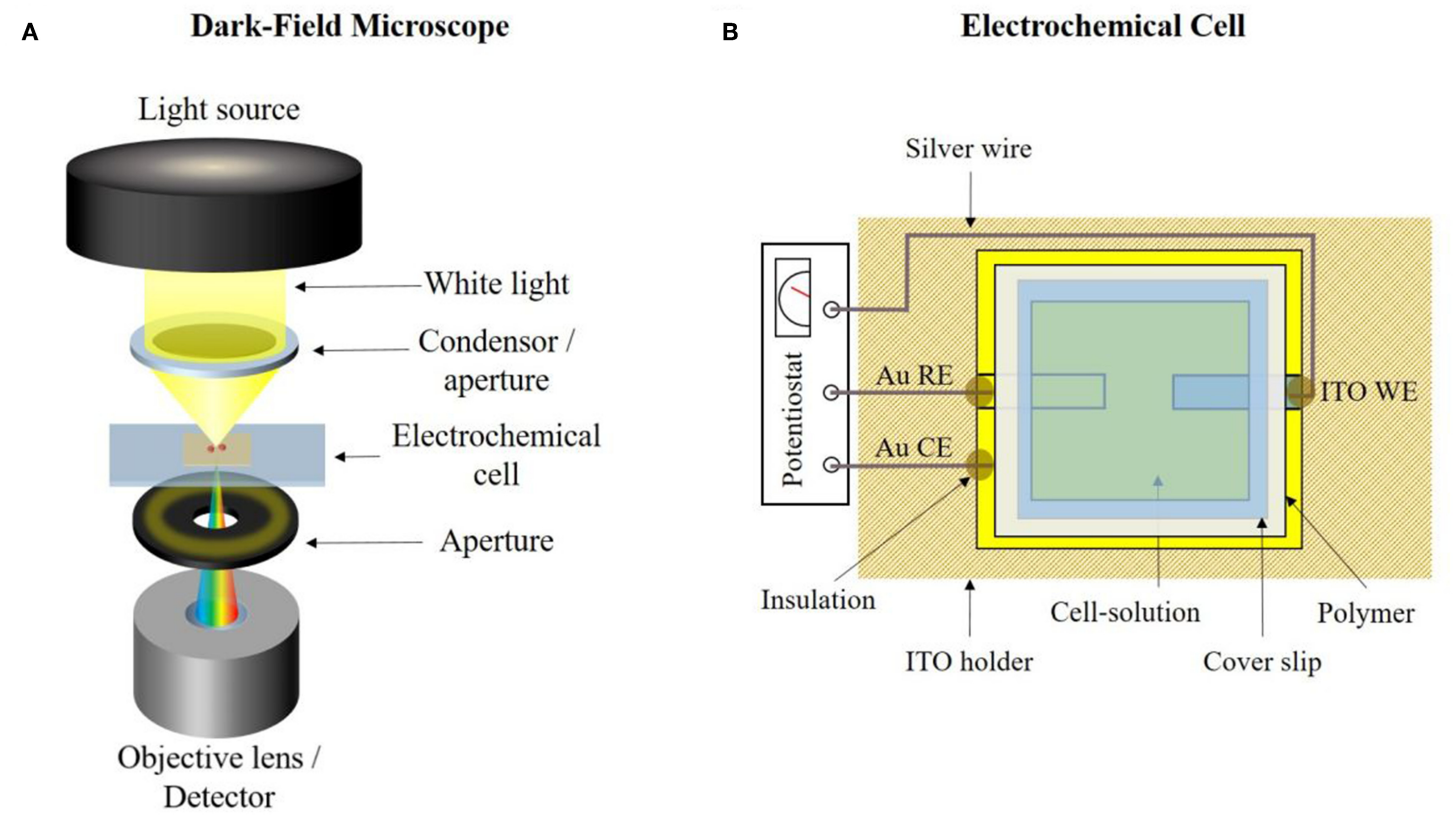
**(A)** Principle setup of a dark-field microscope. **(B)** Designed transparent electrochemical cell for simultaneous opto- and spectro electrochemical dark-field microscopy.

The general working principal of the dark-field microscope is shown in Figure 3A. White light is generated by a halogen lamp and then partly blocked by an aperture and focused by a condenser toward the sample (electrochemical cell). Incoming light interacts with the sample and non-scattered light is blocked by an additional aperture. Only scattered light reaches the objective lens. The light is detected by either an optical charge coupled device (CCD) camera or a hyperspectral camera. The used dark-field microscope was constructed by *CytoViva Inc*. and includes an *Olympus BX43 Microscope*. A *UPL Fluorite 100x/.6-1.30 Iris Objective* oil immersion objective was equipped with an illumination area of 87.5 ·65 μm. Immersion oil ensured a contact between the condenser, sample and oil objective. For illumination, a halogen lamp and a *CytoViva Optical Illuminator* were used. A color *Retiga R1 OEM* Camera (CCD) was used for optical video imaging and a *CytoViva Imaging System 2.5*, consisting of a *Specim ImSpector V10E Spectograph* and a *Cooke Pixelfly Spectral Camera*, was used for hyperspectral imaging. All obtained spectra are wavelength corrected against the halogen lamp spectrum. An optical resolution down to 10–15 nm of plasmon active particles like silver nanospheres can be achieved.

All electrochemical experiments were performed with a *PalmSens* 4 (*PalmSens BV)* potentiostat. For the electrochemical experiments combined with dark-field microscopy, an in-house designed transparent electrochemical cell was prepared. The schematic illustration of this cell is given in Figure 3B. A three-electrode system was used in each measurement, consisting of an ITO working electrode (WE), a gold counter electrode (CE) and a gold quasi-reference electrode (RE). All given potentials were measured against the latter one. For fabrication, a thin gold layer was sputtered on the ITO glass slide using a *Sputter Coater (108auto* from *Cressington Scientific Instruments UK)*. The conductive film between each electrode was mechanically removed. The ITO-glass slide was placed into a PLA-holder and silver wires were used for electrical connection to the electrodes. Epoxy resin was used as insulation and a polymer gasket was placed on the ITO-glass slide to seal the cell. 0.3 μL of the silver nanoparticles were added to the ITO WE and the particles were immobilized at the electrode and remaining liquid was removed with a tissue paper. Then 30–60 μL of electrolyte solution was added and the cell was closed by a cover slip.

## Results

### Hyperspectral Dark-Field Imaging

**Figure 4:**
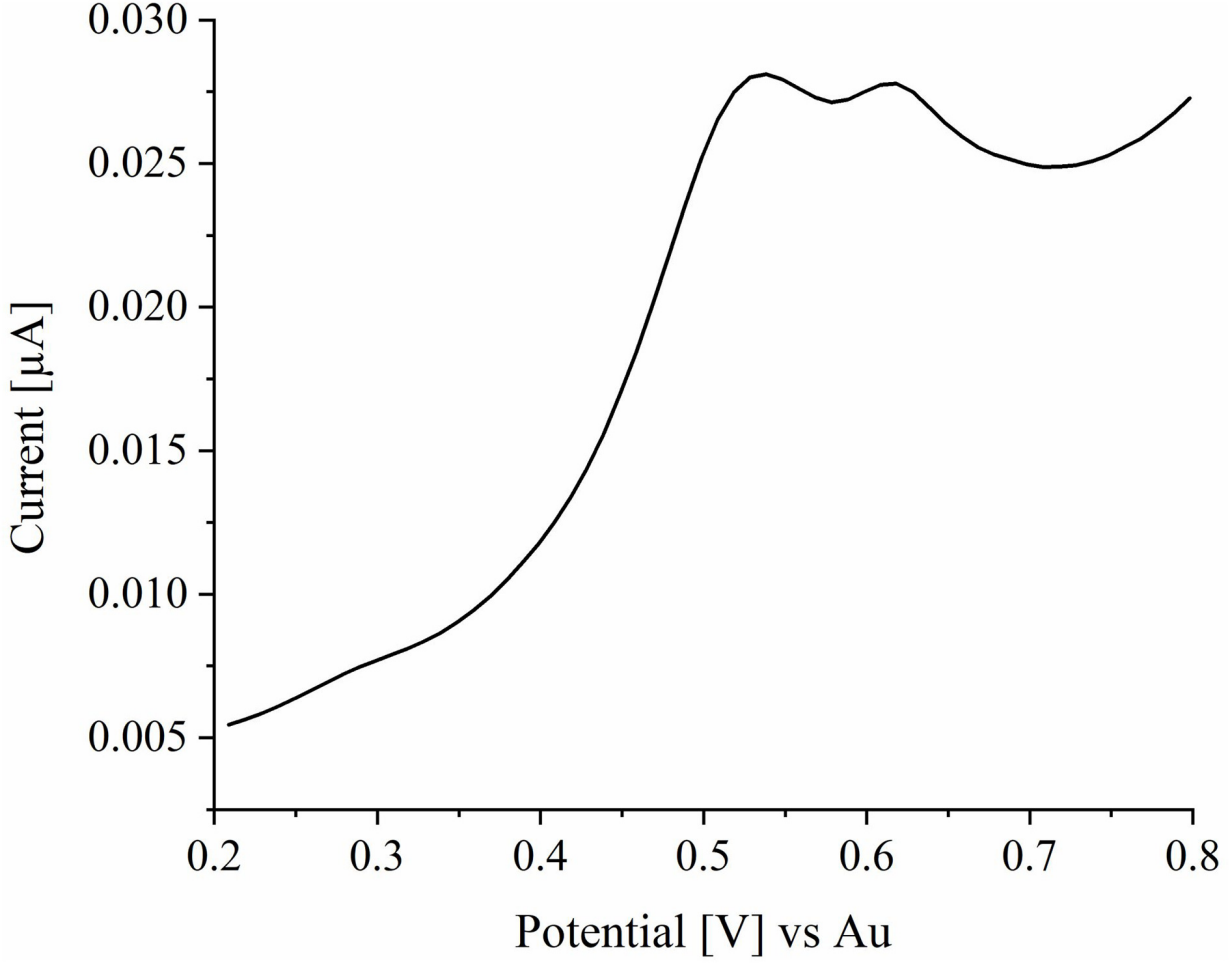
LSV of silver nanoparticles in a 250 mM potassium nitrate solution. The potential was swept from 0.2 to 0.8 V with a scan rate of 0.005 V·s^−1^.

For the oxidation of metallic silver nanoparticles to solvated silver cations, linear sweep voltammetry was performed in a 250 mM potassium nitrate solution. AgNPs (0.3 μL) were immobilized on the ITO surface and electrolyte solution was added. The potential at the ITO WE was swept from 0.2 to 0.8 V with a scan rate of 0.005 V·s^−1^. The corresponding current response plotted over the time is given in the linear sweep voltammogram (LSV) in Figure 4. As it can be seen from the LSV, an oxidation of the silver is observed starting from a potential of 0.45 V. The peak potential is reached at a value of 0.54 V and oxidation is completed at a potential of about 0.72 V. Here, two peaks can be seen. The first peak at 0.54 V can be assigned to the oxidation of silver nanoparticles. The second peak can be assigned to the ITO substrate, as it is also seen in the first cycle of blank experiments in the absence of nanoparticles, represented in Supplementary Figure S1.

**Figure 5:**
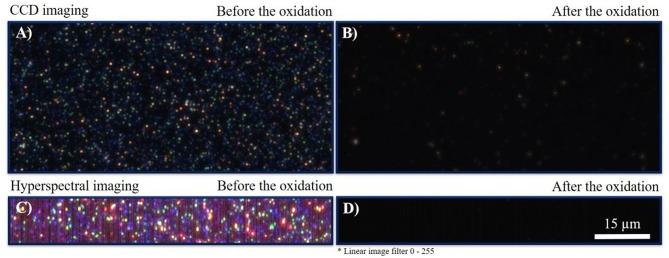
**(A)** DFM-CCD image of AgNPs before the reaction at an exposure time of 2,000 ms. **(B)** DFM-CCD image of AgNPs after the reaction at an exposure time of 2,000 ms. **(C)** HSI-DFM image of AgNPs before the reaction at an exposure time of 1,000 ms. **(D)** HSI-DFM image of AgNPs after the reaction at an exposure time of 1,000 ms. The scale bar is identical for all images.

The comparison of electrochemically obtained current signals with optical CCD dark-field imaging (CCD-DFM) was performed before and after the linear sweep voltammetry measurement. The respective optical snapshots are given in Figures 5A,B. The images were taken at an exposure time of 2,000 ms and an objective magnification of 100. From the images it can be seen that particles are homogeneously distributed and immobilized on the ITO electrode (Figure 5A). After oxidation, most of the nanoparticles disappeared, which is indicated by the complete loss in intensity and color information. From the almost dark image, it can be concluded that the particles fully dissolved during their electrochemical oxidation, while solvated silver cations were formed. Remaining particles are likely not in electrical contact with the ITO electrode (Batchelor-McAuley et al., 2014).

For additional insights, HSI-DFM was performed before and after the reaction. In hyperspectral imaging, single scattering spectra are recorded for each pixel. Based on the spectral information, a color is displayed from a single spectrum. The resulting images are shown in Figures 5C,D and were taken with an exposure time of 1,000 ms. It is indicated that the particles completely vanished and could not be detected. Thus, it is confirmed that the particles dissolved during the oxidation process and did not form a less efficiently scattering insoluble silver species, such as silver oxides (Sundaresan et al., 2018). The optical and hyperspectral images in the absence of silver particles are given in Supplementary Figure S2.

**Figure 6:**
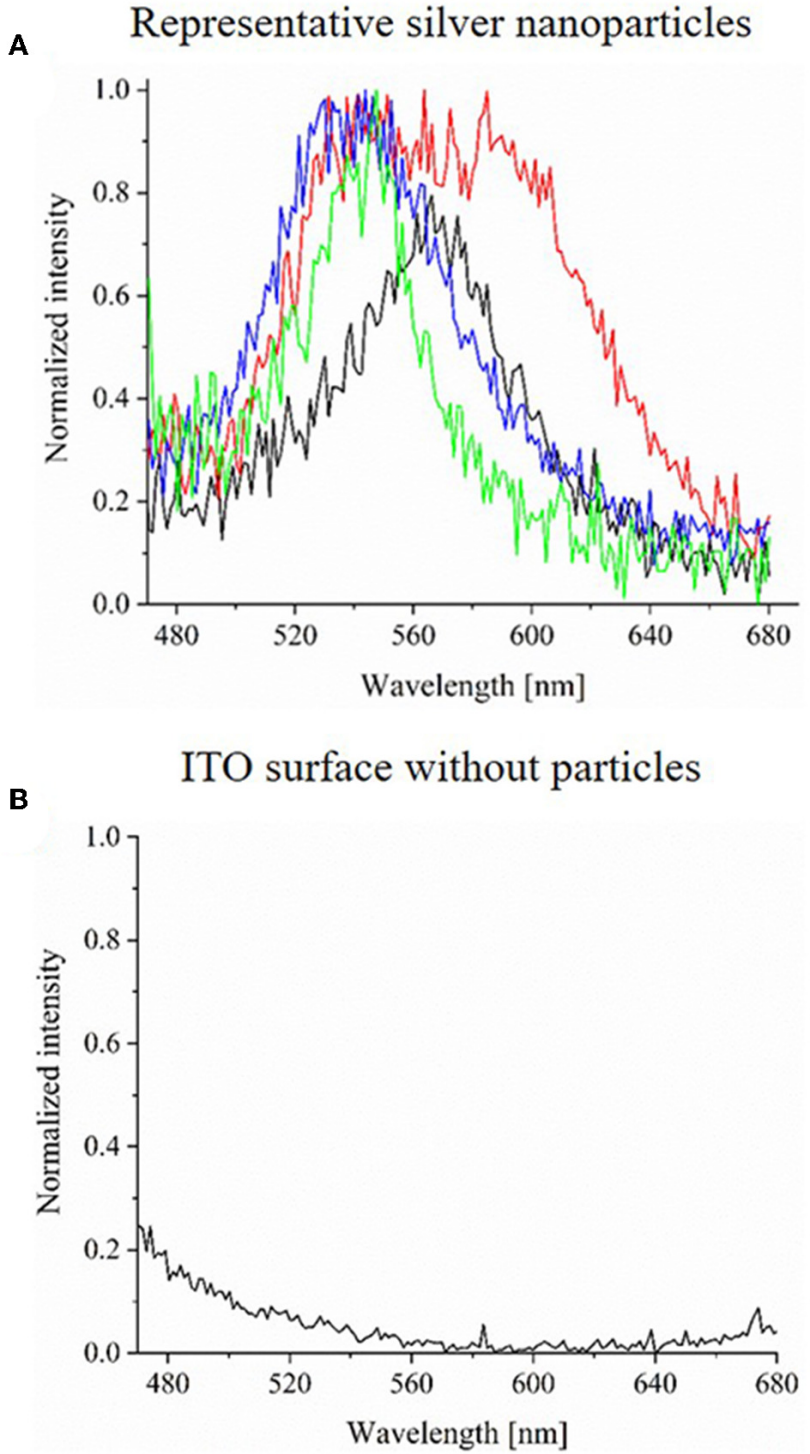
**(A)** Normalized extinction spectra (exposure time of 1,000 ms) of four representative AgNPs before their electrochemical oxidation on ITO. **(B)** Normalized extinction spectrum (exposure time of 1,000 ms) of ITO WE without AgNPs.

Spectra of single silver nanoparticles can be determined by an extraction of the extinction spectra from the HSI. Spectra of four representative AgNPs are plotted in Figure 6A. All intensities were normalized. As it can be observed, all extinction spectra show a peak maximum at wavelengths between 520 and 600 nm. The blank spectrum of the ITO WE without particles is shown in Figure 6B and lacks this specific peak. Furthermore, also the ITO WE in the absence of silver nanoparticles (Supplementary Figure S3) shows no characteristic peaks or shifts during the electrochemical experiment. From these spectra, it is evident that the characteristic LSPR based extinction peak of the particles vanished during the reaction. Nearly all particles were dissolved during the oxidation, which was shown both by the loss of silver particles in CDD- and HSI and by the corresponding spectral changes.

**Figure 7:**
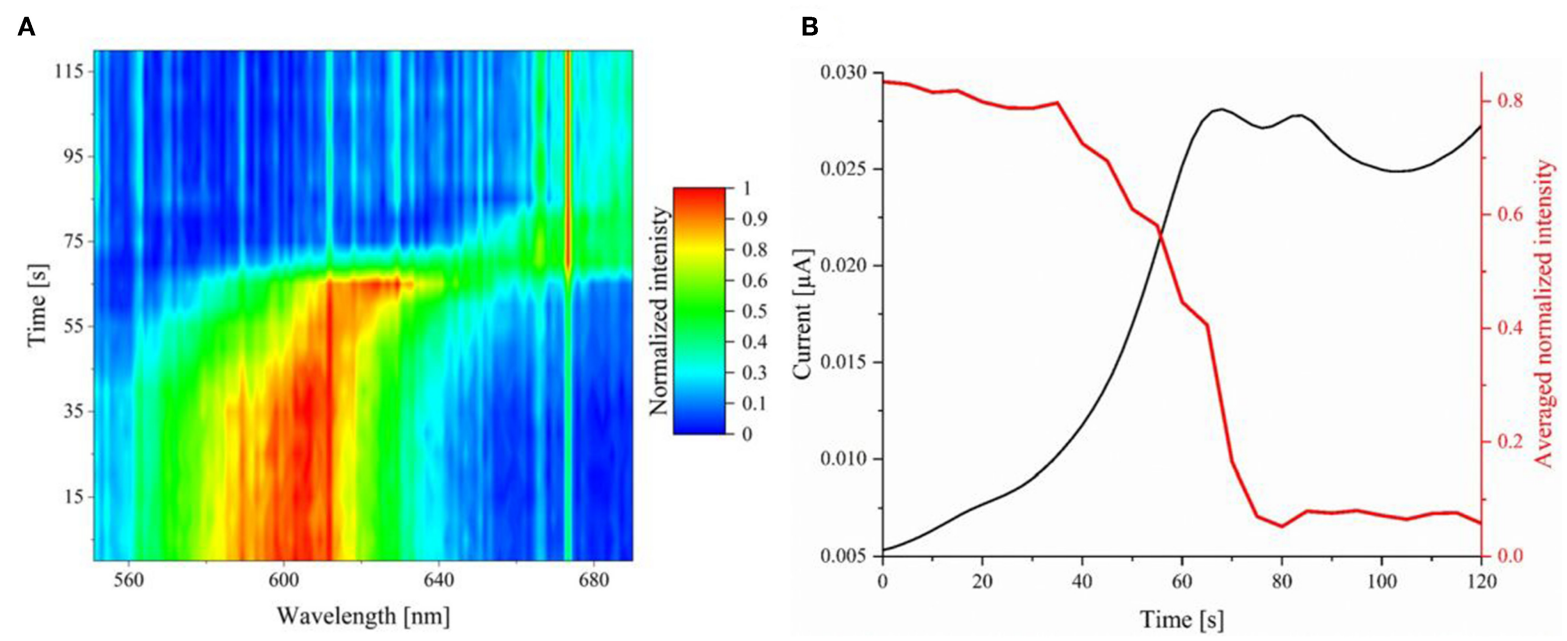
**(A)** Normalized extinction spectra (exposure time of 5 s) of an individual AgNP during its electrochemical oxidation on ITO plotted against the time. **(B)** Averaged and normalized hyperspectral intensity trace (red) and current (black) correlation plotted against the time.

The reaction process of a single silver nanoparticle during the linear sweep voltammetry can be tracked by HSI. To this end, an individual AgNP was tracked at an exposure time of 5 s and an objective magnification of 100. The normalized intensities were plotted against the time in a contour plot, which is given in Figure 7A. The corresponding 3-D illustration of the spectral trace can be found in Supplementary Figure S4. In the intensity trace, it is shown that no spectral change is observed during the first 40 s of the reaction. In the range of 40–45 s small changes in the spectra are obtained.

In addition to the spectral shift, the averaged intensity of wavelengths between 580 and 600 nm was plotted in correlation to the current response over time. The graph is represented in red in Figure 7B, the current is displayed in black.

**Figure 8:**
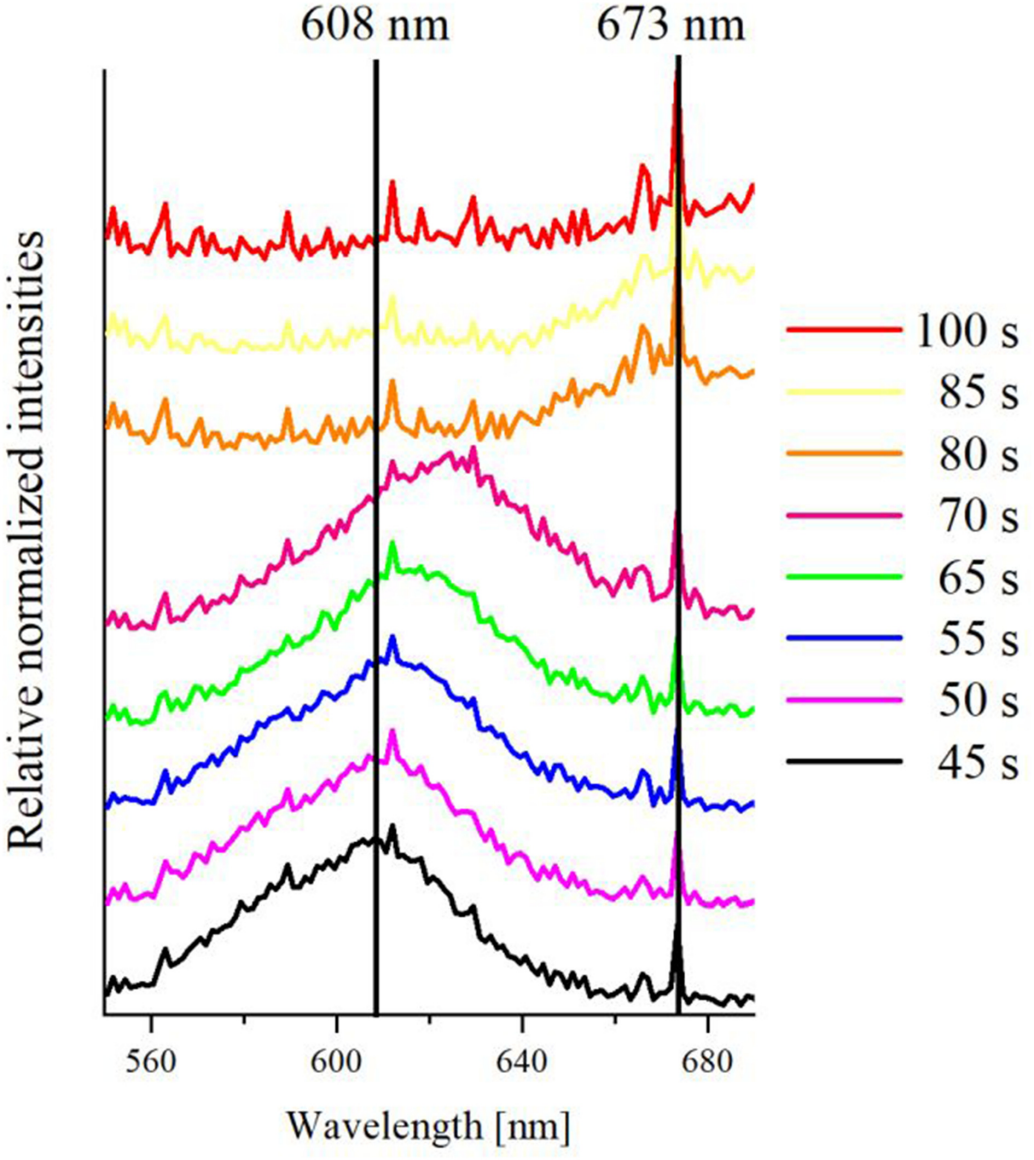
Representative relative and averaged extinction spectra at various times between 45 s and 100 s of a single silver nanoparticle at an ITO WE during linear sweep voltammetry, vertically shifted for better comparability.

It is evident, that there is an intensity decrease staring at 40 s, which is in agreement with the spectral shift and the current response. Regarding the spectral intensity information and the current response, the oxidation is finished at a time of 80–85 s. Notably, current response and intensity trace show an exact inverse shape. This demonstrates that electrochemical methods and spectral observation can be directly compared and lead to conclusive results. From the hyperspectral *operando* imaging it can be concluded that the oxidation and the corresponding dissolution mechanism do not happen instantaneously, but slowly over a longer period of time. For further clarification of the process, representative spectra during the oxidation at experimental times of 45–100 s were plotted against each other with relative (shifted) normalized intensities. The resulting graph is presented in Figure 8.

Two vertical lines at wavelengths of 608 and 673 nm were added as a guide to the eye of spectral changes. As it can be seen, there is a peak maximum of the extinction spectrum at 608 nm at 45 s (black curve), which is based on LSPR light scattering of the initial silver nanoparticle. This peak maximum shifts to 625 nm at 65 s (green curve), while the transition happens over peak maxima at 610 nm (violet curve) and 612 nm (blue curve) at 50 and 55 s, respectively. This indicates that the particle LSPR changed during the oxidation. It can be assumed that this shift happens due to the change of the particle size/shape. The extinction peak of the LSPR after 70 s (pink curve) was extremely shifted to 673 nm, broadened and lost most of its scattering intensity. This can be understood as an ongoing dissolution and shrinkage of the particle. After 80 s (orange curve), the peak shifted to a peak maximum of 673 nm, which is nearly completely vanished at 85 s (yellow curve). After 100 s (red curve), no characteristic peak was detected anymore and only the spectrum of the blank ITO glass (Figure 6B) is observed. Since all spectral information were lost, a full conversion coupled with a total dissolution of an individual silver nanoparticle can be concluded. The control experiments were performed in the absence of silver nanoparticles. The blank cyclic voltammogram and the corresponding normalized extinction spectra against the time are given in Supplementary Figures S1, S5, respectively. No spectral change of the scattering was observed, which further confirms the signal change to be caused by the silver particle dissolution. Nevertheless, a small peak at roughly 0.7 V can be seen in the CV. This can be correlated to the oxidation of the ITO during the first cycle and slightly overlay with the oxidation signal of the silver.

### Optical Dark-Field Imaging

**Figure 9:**
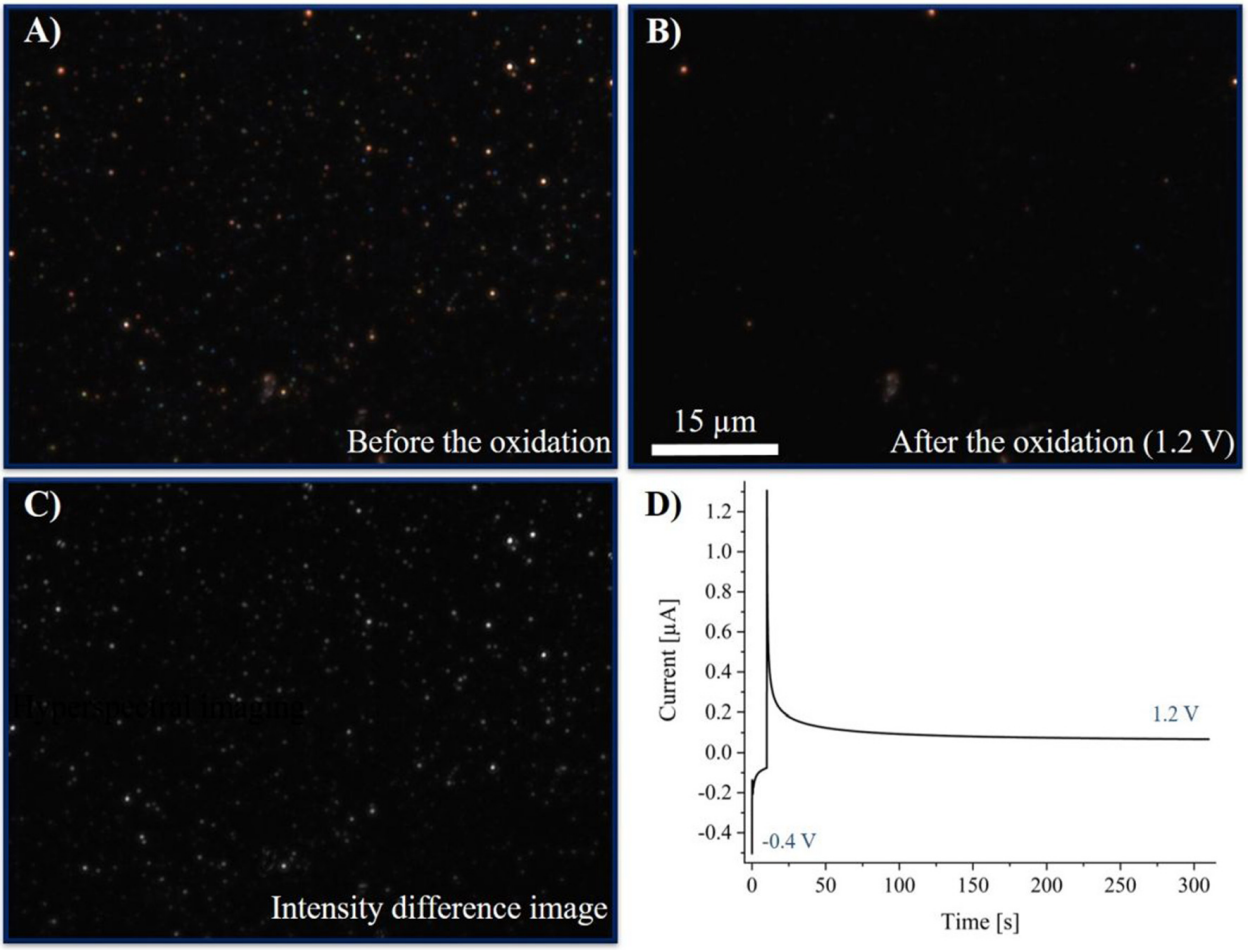
**(A)** DFM-CCD image of AgNPs before the oxidation at an exposure time of 500 ms. **(B)** DFM-CCD images of AgNPs after the oxidation at an exposure time of 500 ms. **(C)** Difference image of **(A,B)**. **(D)** Chronoamperogram of silver nanoparticles in a 250 mM potassium nitrate solution. The potential was held at −0.4 V for 10 s, then the potential was switched to 1.2 V for 300 s.

For comparison of the real-time HSI with shorter exposure times, the reaction was observed with optical CCD-imaging. The results are shown in Figure 9.

Optical images were taken before and after the reaction and are given in Figures 9A,B, respectively. Images were taken at an exposure time of 500 ms and a magnification of the objective of 100. An optical CCD-video was recorded during the reaction with an exposure time of 500 ms, which can be found in Supplementary Material Video 1 and is represented with 20 fps. A multistep chronoamperometric experiment was performed, starting with an applied potential of −0.4 V for the first 10 s and a subsequent switch to 1.2 V for 300 s. The obtained chronoamperogram (current-time plot) is plotted in Figure 9D. The corresponding chronoamperogram, optical images before and after the electrochemical experiment and the video imaging of the electrochemical experiment in the absence of silver nanoparticles are given in Supplementary Figures S6, S7 and Supplementary Material Video S1, respectively.

Previous to the electrochemical experiment, the AgNPs were immobilized as elemental nanoparticles on the electrode. Hence, no change in the color and thus in the LSPR was observed while a potential of −0.4 V was applied. From the chronoamperogram, only the negative double layer charging current was observed during the first 10 s. Changing the potential to 1.2 V, a slight decrease in intensity and a simultaneous color change of the particle images was detected. Moreover, a simultaneous steep increase in the current to positive values was obtained. This indicates on the one hand the oxidative current based on the oxidation of AgNPs and on the other hand the inverted double layer charging. As it can be seen in the video, the dissolution does not take place instantaneously, but over several measurement frames. Hence, it can be concluded, that the particle dissolution occurs rather slowly and might happen stepwise.

Optical and hyperspectral results are in accordance with each other. The created difference image (Figure 9C) shows in advance, that nearly all of the particles are dissolved during the reaction. As it can be seen in Figure 9B, a minority of particles (or impurities) was left after the reaction, which might have lacked electrical contact or did not consist of silver. However, these features can be used as an internal reference to ensure that no significant drift of the focus, and, hence, of the detected intensity of imaged particles occurs within the time scale of the experiment. Thus, this change in the extinction of the nanoparticles proves the disappearing of the other AgNPs during the reaction, which otherwise might have arisen from a change in focus of the system.

**Figure 10:**
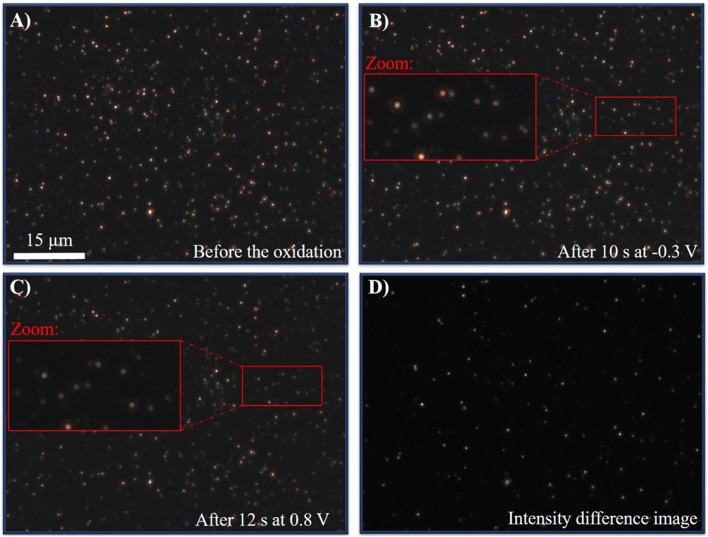
**(A)** DFM-CCD image of AgNPs before the oxidation at an exposure time of 100 ms. **(B)** DFM-CCD images of AgNPs after 10.0 s at −0.3 V at an exposure time of 100 ms. **(C)** DFM-CCD images of AgNPs after 10.2 s at 0.8 V at an exposure time of 100 ms. **(D)** Difference image of **(B,C)**.

To validate that the reaction in nitrate electrolytes is indeed slow and not decelerated by the experimental setting (e.g., the resistivity of the ITO WE), a different electrolyte was tested with complexing counter ions. Because silver is preferentially forming AgCl in the presence of chloride anions (Equation 2), the reaction should be performed in a chloride containing electrolyte of equal ionic strength. In previous works it was shown that silver nanoparticles react nearly instantaneously in 50 mM KCl on a platinum electrode (Wonner et al., 2018). In this work, a 250 mM solution of KCl_(aq)_ was used. A multistep chronoamperometric experiment was performed as before with an initial potential of −0.3 V for 10 s and 0.8 V for 20 s. Optical CCD-imaging was performed during the reaction with an exposure time of 100 ms. Snapshots of the immobilized AgNPs before the electrochemical measurement (A), after 10.0 s at −0.3 V (B), and after 12 s at 0.8 V (C) are given in Figures 10 A–C, respectively. The optical video imaging is given in the Supplementary Material Video 2. The corresponding chronoamperogram in the presence and absence of silver nanoparticles is displayed in Supplementary Figures S8, S9, the video imaging in the absence of silver particles in chloride containing solutions is given in Supplementary Material Video S2, respectively.

From the video (0–10 s) and the images Figures 10A,B it is evident that no color or intensity change can be observed during the first 10 s, when a potential of −0.3 V was applied. This indicates that elemental silver nanoparticles were immobilized at the surface, which cannot be reduced further. Switching to an oxidative potential of 0.8 V (10–30 s in the video and Figure 10C, a clear, distinct and instantaneous change in the scattering intensity and color change was observed. This clear and fast shift indicates the oxidation to silver chloride. Comparing the scattering of the particles after 12 s (Figure 10C) and 10 s (Figure 10B), a difference image of both states can be created. The resulting difference image is given in Figure 10D. As it can be seen, most of the particles changed their appearance due to the oxidation, which is represented as bright dots in the image. Hence, it can be concluded that within 2 s most of the particles reacted. This is in agreement with previous studies in chloride solution (Wonner et al., 2018) and much faster than the timescale of the reaction in nitrate containing electrolyte, discussed above.

With this control experiment, it was shown that the oxidation of silver in chloride is not hindered by, for example, insufficient potential drop at the ITO WE. Compared to the dissolution in nitrate, the oxidation in chloride is accelerated and finished after a short time. Hence, it can be concluded that the reaction and coupled dissolution of silver nanoparticles is indeed slowed down in the presence of nitrate anions. Nitrate as a non-complexing counter ion requires the oxidation to solvated silver cations in the solution, which is identified as a significantly slower process in this work that the electrochemical oxidation of silver to silver chloride.

## Discussion

In this work, it was shown that individual immobilized silver nanoparticles can dissolve during electrochemical oxidation in a nitrate containing electrolyte. It was observed that the particles vanished completely after a reaction times of several seconds. Furthermore, the dissolution process seems to be a slow process, compared to the oxidation in the presence of chloride anions. The deceleration of the oxidation process seems to be based on the thermodynamic unfavorable silver cation formation in the presence of non-complexing counter ions, in contrast to the silver chloride formation in the presence of precipitating anions.

This study gives insight into the reaction dynamics of individual nanoparticles during their electrochemical oxidation. It can be considered that the oxidation process in nitrate is not an instantaneous and full dissolution, but rather a slow dissolution mechanism. The results give hints that the oxidation during single nanoparticle impacts at a polarized electrode happen over several successive events. Nevertheless, neither a *hopping* nor a movement of the particles, which was already assumed in literature (Oja et al., 2017; Ustarroz et al., 2017) was observed.

Based on this study it is evident that *operando* opto- and spectro-electrochemical dark-field microscopy studies are a strong tool to characterize processes at individual nano entities and differentiate the reaction dynamics in various systems. Accordingly, it is anticipated that this approach may be exploited systematically to study electrocatalytic processes at different nanomaterials in the future.

## Data Availability Statement

The raw data supporting the conclusions of this manuscript will be made available by the authors, without undue reservation, to any qualified researcher.

## Author Contributions

All authors listed have made a substantial, direct and intellectual contribution to the work, and approved it for publication.

### Conflict of Interest

The authors declare that the research was conducted in the absence of any commercial or financial relationships that could be construed as a potential conflict of interest.

## Supplementary Material

3-D HSI plot, Chronoamperogram in the presence of silver nanoparticles in potassium chloride solution, Optical and hyperspectral imaging in the absence of silver nanoparticles, LSV and chronoamperogram's in the absence of silver nanoparticles (PDF).

Supplementary Video 1 The video shows the dissolution process of silver nanoparticles during its electrochemical oxidation in 250 mM KNO_3_ at an ITO electrode at a potential of −0.4 V for 10 s and 1.2 V for 300 s. Images were taken at an exposure time of 500 ms and an objective magnification of 100.

Supplementary Video 2 The video shows the reaction of silver nanoparticles during its electrochemical oxidation in 250 mM KCl at an ITO electrode at a potential of −0.3 V for 10 s and 0.8 V for 20 s. Images were taken at an exposure time of 100 ms and an objective magnification of 100.

Supplementary Video S1 The video shows the blank experiment in the absence of silver nanoparticles in 250 mM KNO_3_ of an ITO electrode at a potential of −0.4 V for 10 s and 1.2 V for 300 s. Images were taken at an exposure time of 500 ms and an objective magnification of 100.

Supplementary Video S2 The video shows the blank experiment in the absence of silver nanoparticles in 250 mM KCl of an ITO electrode at a potential of −0.3 V for 10 s and 0.8 V for 300 s. Images were taken at an exposure time of 100 ms and an objective magnification of 100.
