# Supplementary material for: Operando Studies of the Electrochemical Dissolution of Silver Nanoparticles in Nitrate Solutions Observed With Hyperspectral Dark-Field Microscopy
Source: Front Chem. 2020 Jan 17;7:912. doi: 10.3389/fchem.2019.00912 (PMC6978802; doi:10.3389/fchem.2019.00912)
Supplement: Supplementary file 2 [file Data_Sheet_2.PDF]

Supplementary Material

Operando Studies of the Electrochemical Dissolution of Silver Nanoparticles in Nitrate Solutions Observed with Hyperspectral Dark-Field Microscopy

Kevin Wonner[1], Christian Rurainsky[1], Kristina Tschulik[1*]

[1]Ruhr University Bochum, Faculty of Chemistry and Biochemistry, Chair of Analytical Chemistry II, Bochum, Germany

*** Correspondence:**
Prof. Dr. Kristina Tschulik
Kristina.tschulik@rub.de

1 Supplementary Figures

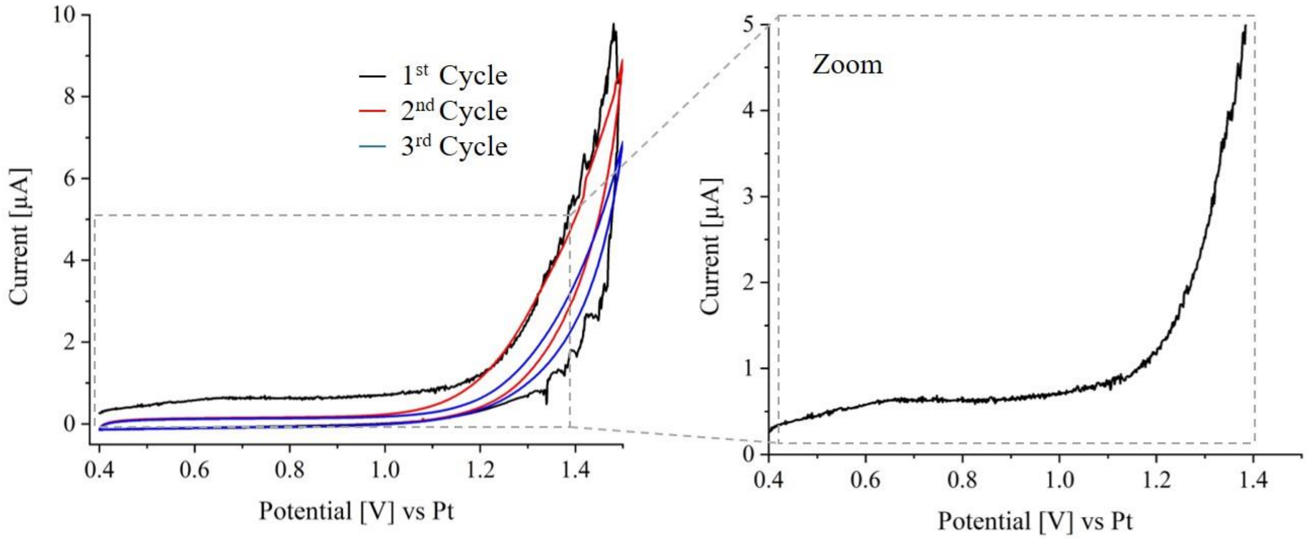

Supplementary Figure S1: Left**:** CVs recorded in the absence of silver nanoparticles in a 250 mM potassium nitrate solution show no specific oxidation peak of silver in three subsequent cycles, but a small oxidation peak at about 0.7 V in the first cycle. The potential was swept between 0.4 V to 1.5 V with a scan rate of 0.025 V s^{-1}.Right: Zoom of the first cycles between 0.4 V and 1.4 V.

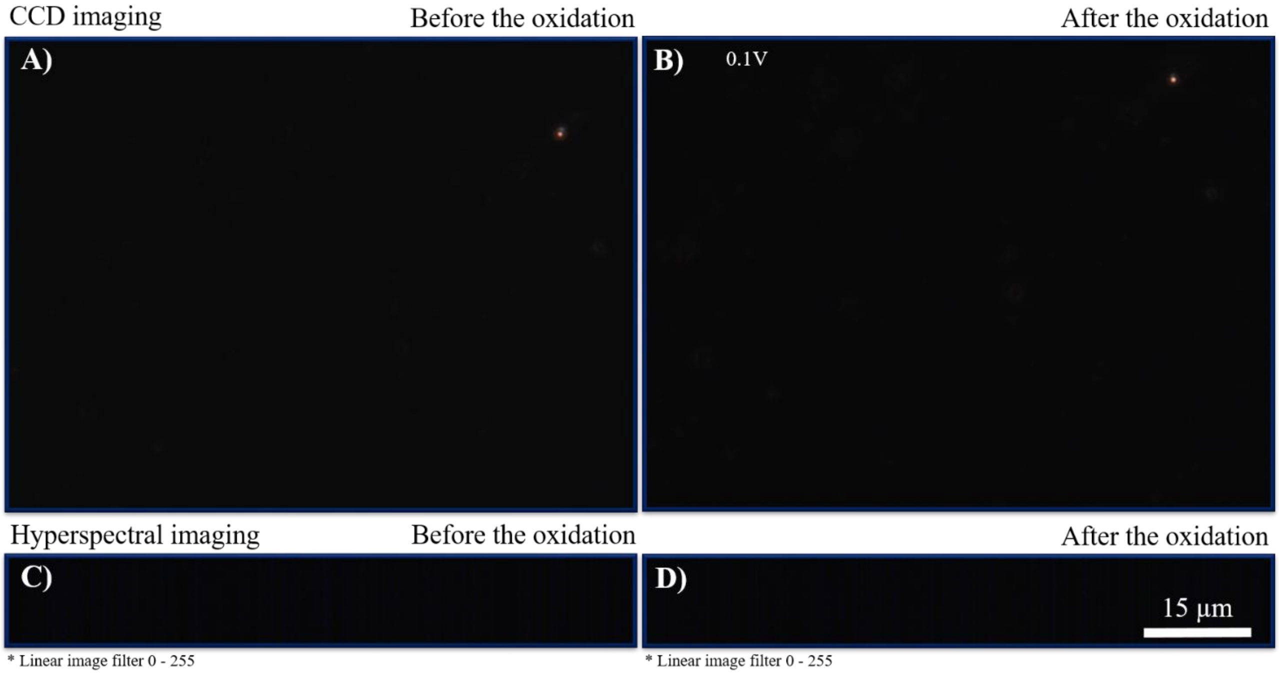

Supplementary Figure S2: A) DFM-CCD image of the ITO WE before the linear sweep voltammetry (LSV) experiment at an exposure time of 2000 ms in the absence of silver nanoparticles. B) DFM-CCD image of the ITO WE after the LSV experiment at an exposure time of 2000 ms in the absence of silver nanoparticles. C) / D) HSI-DFM image of the ITO WE in the absence of silver nanoparticles before (C)) the reaction and after the LSV experiment (D)) at an exposure time of 1000 ms. The scale bar is identical for all images.

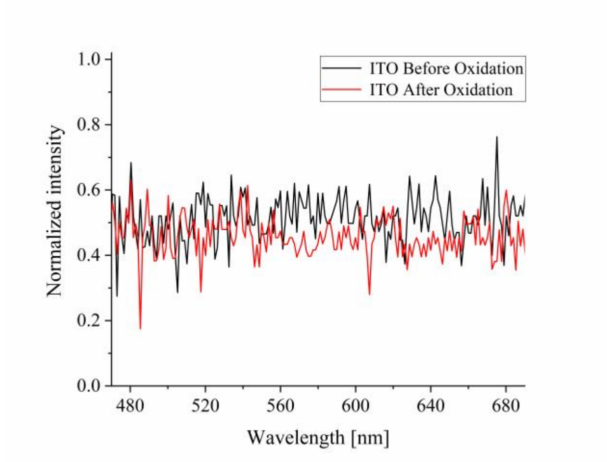

Supplementary Figure S3: Normalized extinction spectra (exposure time of 1000 ms) of the ITO WE before and after the electrochemical experiment in the absence of silver nanoparticles.

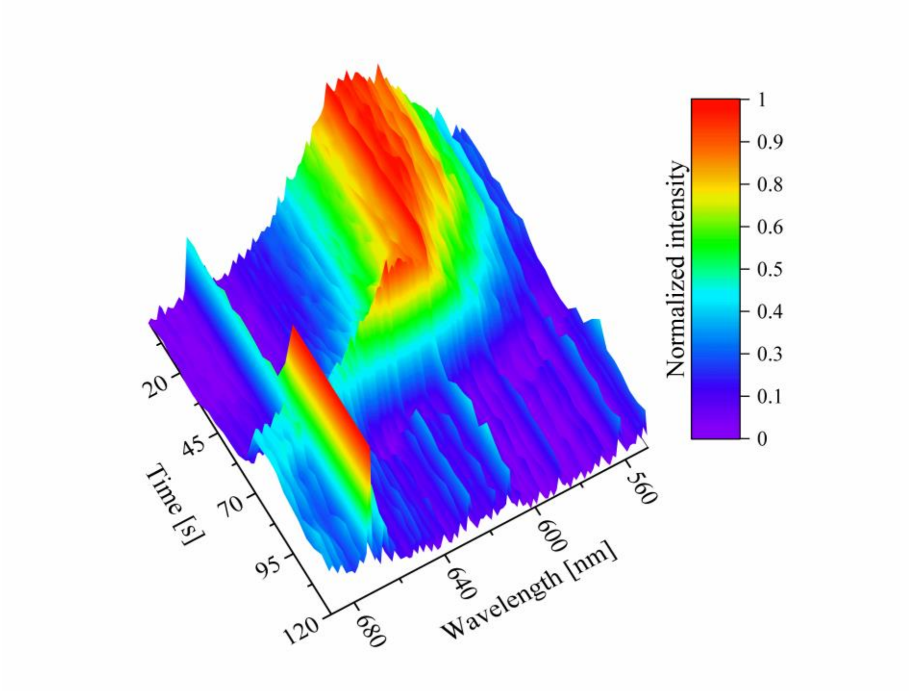

Supplementary Figure S4:3-D representation of the normalized extinction spectra (exposure time of 5 s) of an individual AgNP during it electrochemical oxidation on ITO plotted against the experimental time. The potential was swept from 0.2 V to 0.8 V with a scan rate of 0.005 V s^{-1} and shows the spectral trace and shift of the extinction spectrum of a single nanoparticle during its electrochemical dissolution in nitrate solution.

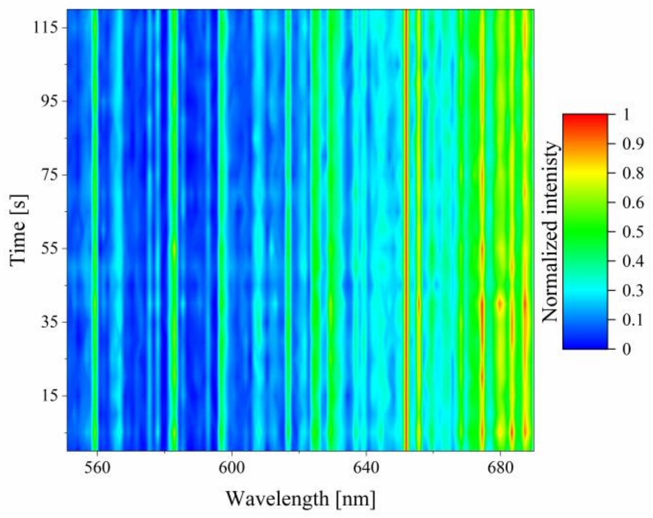

Supplementary Figure S5: Normalized extinction spectra (exposure time of 5 s) in the absence of silver nanoparticles during their linear scan on ITO plotted against the time does not show specific changes and no characteristic spectral peak, which would be recorded for an AgNP. The potential was swept from 0.2 V to 0.8 V with a scan rate of 0.005 $V \cdot s^{-1}$.

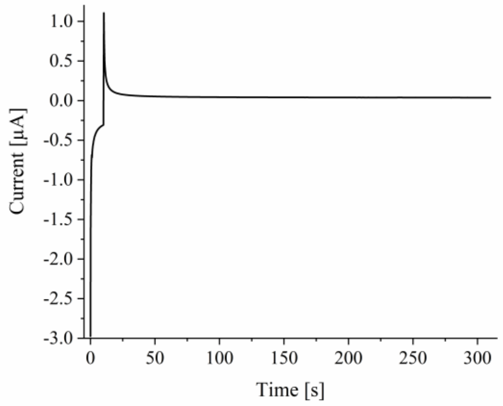

Supplementary Figure S6: Chronoamperogram in the absence of silver nanoparticles in a 250 mM potassium nitrate solution. The potential was held at -0.4 V for 10 s. The potential was stepped to 1.2 V for 300 s.

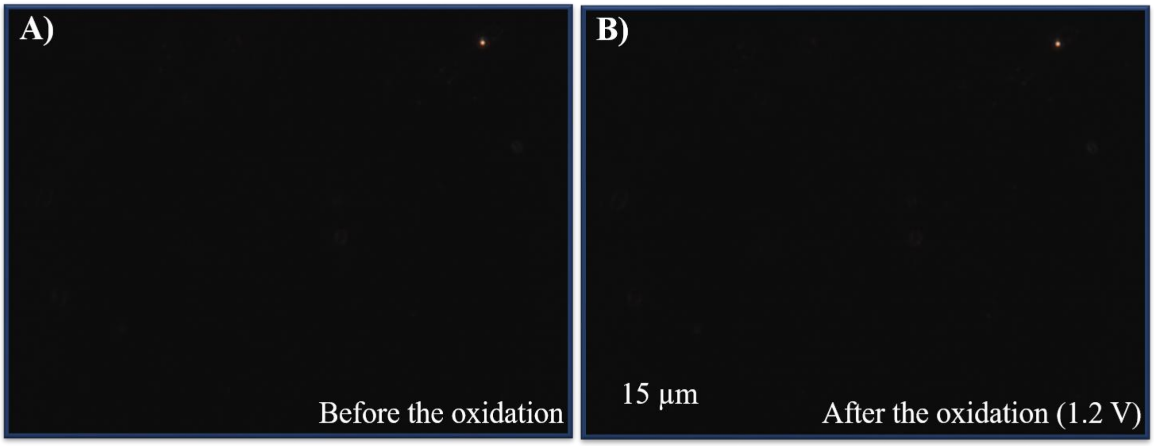

Supplementary Figure S7: A) DFM-CCD image of the ITO WE in the absence of silver nanoparticles before the oxidation at an exposure time of 500 ms. B) DFM-CCD images of the ITO WE in the absence of silver nanoparticles after the oxidation at an exposure time of 500 ms.

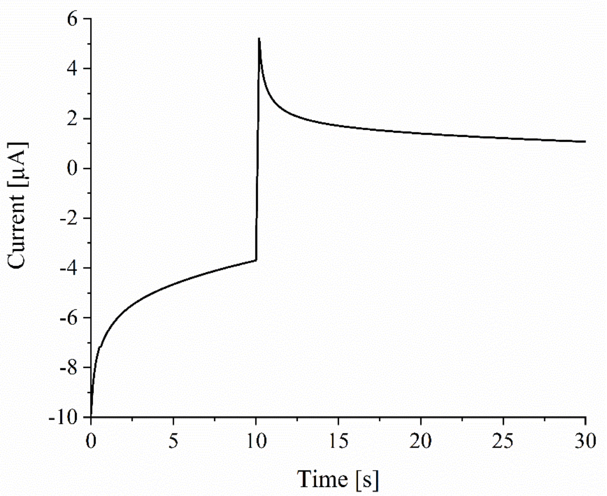

Supplementary Figure S8: Chronoamperogram of silver nanoparticles in a 250 mM potassium chloride solution. The potential was held at -0.3 V for 10 s. The potential was stepped to 0.8 V for 20 s.

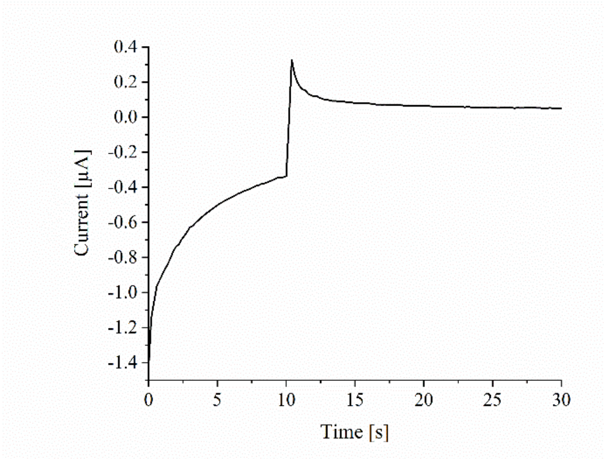

Supplementary Figure S9: Chronoamperogram in the absence of silver nanoparticles in a 250 mM potassium chloride solution. The potential was held at -0.3 V for 10 s. The potential was stepped to 0.8 V for 20 s.
